# An Evolving Technology That Integrates Classical Methods with Continuous Technological Developments: Thin-Layer Chromatography Bioautography

**DOI:** 10.3390/molecules26154647

**Published:** 2021-07-31

**Authors:** Meng Wang, Yirong Zhang, Ruijie Wang, Zhibin Wang, Bingyou Yang, Haixue Kuang

**Affiliations:** Key Laboratory of Chinese Materia Medica (Ministry of Education), Heilongjiang University of Chinese Medicine, Harbin 150000, China; wangmeng@hljucm.net (M.W.); 18845032923@163.com (Y.Z.); wangruijie170@163.com (R.W.); wzbmailbox@126.com (Z.W.); ybywater@163.com (B.Y.)

**Keywords:** thin-layer chromatography, bioautography, segregation analysis, activity detection, practical application

## Abstract

Thin-layer chromatography (TLC) bioautography is an evolving technology that integrates the separation and analysis technology of TLC with biological activity detection technology, which has shown a steep rise in popularity over the past few decades. It connects TLC with convenient, economic and intuitive features and bioautography with high levels of sensitivity and specificity. In this study, we discuss the research progress of TLC bioautography and then establish a definite timeline to introduce it. This review summarizes known TLC bioautography types and practical applications for determining antibacterial, antifungal, antitumor and antioxidant compounds and for inhibiting glucosidase, pancreatic lipase, tyrosinase and cholinesterase activity constitutes. Nowadays, especially during the COVID-19 pandemic, it is important to identify original, natural products with anti-COVID potential compounds from Chinese traditional medicine and natural medicinal plants. We also give an account of detection techniques, including in situ and ex situ techniques; even in situ ion sources represent a major reform. Considering the current technical innovations, we propose that the technology will make more progress in TLC plates with higher separation and detection technology with a more portable and extensive scope of application. We believe this technology will be diffusely applied in medicine, biology, agriculture, animal husbandry, garden forestry, environmental management and other fields in the future.

## 1. Introduction

There are abundant resources of animals, plants and microorganisms in nature, from which the screening and extraction of bioactive substances have always been the focus of research at home and abroad. Chemical constituents in biology are manifold and structure utterly different, but those with biological activity are less proportionate. Therefore, the rapid detecting and screening of biological active components is vital to the study of natural active substances. However, the traditional method of the separation of active ingredients from natural products also needs to pass for isolate compounds in natural products, and the screening of the active compound is performed in two steps. The activity of general appraisal should be carried out on the entire history of the animal or organ, because isolated compounds often appear for the active substance, or the quantity is not enough for a large number of active screenings [1]. Consequently, establishing a rapid and accurate screening method for active substances plays an important role in the separation of active substances, the discovery of new compounds, the control of the quality standards and the expansion of the application range of the components.

TLC bioautography is used to isolate, locate and evaluate the active constituents of natural creatures based on the guidance of activity referring to a methodological technique and has the advantages of a simple operation with low cost and high levels of sensitivity and specificity. This technique combines TLC separation with biological activity determination; TLC is a modern chromatography technology in which mixtures are separated by adsorption materials coated on the surface of the supporting plate as the stationary phase and eluent as the mobile phase. By means of utilizing the different adsorption capacities of each component in the stationary phase, continuous adsorption and desorption can be generated during the mobile phase elution process to achieve the separation of different components. TLC as a classic analytical technique was first developed in 1938. It has advantages of low cost, quick development time, good reproducibility and high sensitivity. With the development of the technology, two-dimensional thin-layer chromatography (2D-TLC) and high performance thin-layer chromatography (HPTLC) were derived on this basis, and the application range of this kind of detection technology is expanding continuously. Natural phospholipids have been quantified by 2D-TLC, which verified the applicability of the technology [2,3]. In 2018, Oksana Sytar et al. studied the content of anthocyanins in the grains and sprouts of colored wheat genotypes by HPTLC [4]. Sathis Kumar Dinakaran et al. used HPTLC and TLC to fingerprint the chemical components of polyherbal products [5]. Bioautography is a method that uses chemical components to react with substrates and chromogenic agents to form disparate color contrasts, so as to observe contrast spots in a chromogenic background to track active components [6]. TLC combined with bioautography can reduce the blindness of compound separation and reduce the use of active animals compared with traditional methodology. On the one hand, it can reflect the difference of chemical composition between samples. On the other hand, this assay format allows preassigning of the biological activity, observed in a mixture, to one or a few of its components through the spots that they produce [7]. It determines the presence, absence or strength of the biological activity of the detected substance by showing spots with different colors from the inactive area under certain conditions, which is especially suitable for the screening and discovery of active ingredients in complex mixture systems.

**Figure 1:**
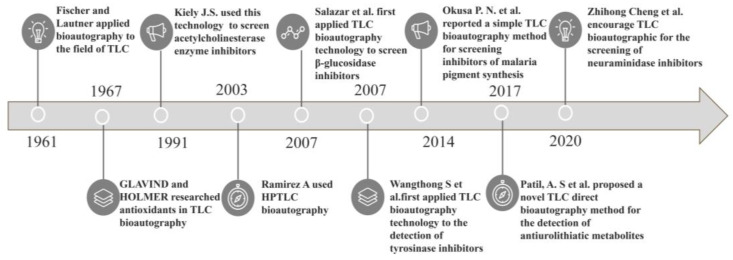
Historical development of TLC bioautography. This figure summarizes the significant experiments in the period starting from the invention of the technique in 1961 to the screening of neuraminidase in 2020.

Over the course of 60 years, the technology has been refined and optimized. The emergence of TLC bioautography can be traced back to 1961, when Fischer and Lautner applied the method to the field of antimicrobials. Thereafter, Glavind and Holmer expanded applications to antioxidants. In 1991, Kiely J.S. used it to screen enzyme inhibitors, and, later, the literature reported that pancreatic lipase inhibitors in the middle have a preeminent behavior on the treatment of obesity and prevention of its complications [8,9,10]. Since December 2019, the infection of the coronavirus disease 2019 (COVID-19) has spread worldwide; COVID-19 is caused by severe acute respiratory syndrome-coronavirus-2 (SARS-CoV-2) and usually starts with influenza-like symptoms. It has many of the same key proteins and targets during transmission and infection as influenza [11]. Neuraminidase is a glycoprotein present in the outermost envelope of influenza viruses; this protein catalyzes the hydrolysis of the glycosidic bonds between sialic acid and glycoproteins [12] and plays an important role in the replication and diffusion of influenza viruses by aiding virus release from the host cells. The in silico analysis of 18 extracted compounds of 11 Indian herbal plants demonstrated different inhibitory properties against COVID-19. The potential inhibitory effect comes from plants such as Nyctanthes arbortristis, Aloe barbadensis miller and Tinospora cordifolia [13,14]. In addition, Chinese traditional medicine (Lianhuaqingwen and ShuFengJieDu capsules) could aid in COVID-19 treatment [15]. Zhihong Cheng et al. encourage the development of a new TLC bioautographic method for the screening of neuraminidase inhibitors to help combat influenza in an epidemic environment [16]. Okusa P. N. et al. reported a simple TLC bioautography method for screening inhibitors of malaria pigment synthesis in 2014 [17]. Five years later, Patil, A. S et al. proposed a novel TLC direct bioautography method for the detection of antiurolithiatic metabolites [18]. The major historical evolution of this technology is shown in Figure 1. With the passage of time, the technology has developed steadily. Furthermore, the biological application of TLC bioautography has been promising and has multiplied, and now it has been diffusely applied in the screening, identification, quality inspection and standard formulation of food, medicine and cosmetics; the analysis of biological samples, poison, chemicals and high polymer material; pest control in animal agriculture husbandry, garden forestry and agriculture; and environmental management, medical science and other fields. Later, the application scope of this technology will inevitably broaden. In view of the scattered previous studies and the lack of comprehensive collation, this paper systematically reviews this technology, analyzes its historical development and collates its biological applications, which will benefit and deepen the understanding of this method and make it possible to have broader applications.

## 2. Classification

As shown below, three basic types of TLC bioautography can be distinguished: agar diffusion, direct bioautography and agar overlay bioautography, and, in addition, HPTLC bioautography and 2D-TLC bioautography, which have emerged in recent years.

### 2.1. Classical Types

#### 2.1.1. Agar Diffusion

Also known as the agar contact method, agar diffusion is also the least-employed one of these techniques. In this method, medium inoculated with pathogenic microorganisms is used as the carrier, and a thin-layer plate adsorbed with the compounds is face down and in contact with the surface of the medium. The compound is placed in contact with the surface of the medium for a certain time to diffuse it, and, then, after removal of the thin-layer plate, the medium is cultured overnight at about 37 °C for bioautography. Agar is prone to adhere to silica gel due to the prominent adsorption between them; therefore, it is easy to damage the agar layer and affect the observation of the spots after removal of the thin plate; thus, adding a wet filter paper between the plate and the agar layer could reduce this error to a certain extent. In addition, another defect of the agar diffusion is that compounds will be lost during the transfer from the thin-layer plate to the culture medium, which is not conducive to the precise determination of the concentration and brings about a low level of sensitivity and specificity, cooperatively ensuring the utilization rate of this method is on the decline.

In recent years, some scholars have used this method in the prevention of diseases and insect pests and achieved certain success. For example, apple ring rot, caused by *Botryosphaeria dothidea*, is a serious apple disease, and researchers found that fengycin-like lipopeptides (retention factor 0.1~0.2), which exhibit antifungal activity against *B. dothidea*, were observed in the *Bacillus subtilis* strain of healthy apples by TLC bioautography analysis. This is the first report on the use of a *B. subtilis* strain as a potential biological control agent to control apple ring rot disease caused by the production of fengycin [19]. We believe this method will be used in more fields in the future.

#### 2.1.2. Direct Bioautography

Direct bioautography (DB) is the most commonly used and easiest method among the five methods. This method, using a thin-layer plate as the carrier, sprays the pathogenic microorganism suspension with a certain concentration of specific nutrient solution directly onto the unfolded thin-layer plate, or directly places the plate into the suspension. It can observe the experimental result directly or under the appropriate color reagent after cultivation in a humid and dark environment for a period of time. In this method, separation, pretreatment, cultivation and development are performed directly on the plate. The action duration of this method is longer, which is conducive to the preservation of the experimental results. This is the simplest method for the detection of antifungal substances, such as *Aspergillus*, *Penicillium* and *Mycobacteria* [20,21]. However, this method has some limitations and is only applicable to microorganisms that can grow normally on thin-layer plates. In practice, in order to evenly distribute pathogenic microorganisms on the thin plate, a spray bottle or roller device can be used to coat the suspension of bacteria or fungi; the latter can minimize the content of the microorganisms floating in the air. The incubation temperature is generally slightly higher than room temperature, and the incubation time is 2~3 days. It can also be cultured overnight at 30 °C or thereabout. Sometimes, light is needed to make the microorganisms enter into a stable growth period [22,23].

DB is widely used in a variety of research such as that of Mary A. Bisi-Johnson et al., who spotted and developed crude extracts from 14 plants and sprayed microbial suspension on a dry chromatographic board in order to test and evaluate the antibacterial ability of the plants. The results showed that *Staphylococcus aureus* and *Shigella flexneri* were the most sensitive isolates to crude extracts and confirmed the antibacterial effect of *Aloe arborescens* Mill. and *Psidium guajava* L on the spectrum of β-lactamase-positive salmonella enterica infection [24]. Viktória Lilla Balázs et al. studied the antibacterial activity of the essential oil extracts and main constituents of clove, cinnamon, thyme and peppermint against *Haemophilus influenzae* and *H. parainfluenzae*. The results showed that thyme and cinnamon oils were the most effective among the investigated oils [25]. With the gradual diversification of bioautography, there is a TLC bioluminescence, which is a variation of bioautography detection in TLC [26]. Although it is not based on changes in bacterial growth but on the fluorescence quenching of *Luminescence bacilli* and *Vibrio fischeri*, in addition to naturally occurring bacteria, some genetically modified bacteria with combined fluorescence genes such as *Acinetobacter* can also be applied.

#### 2.1.3. Agar Overlay Bioautography

Also known as immersion bioautography, agar overlay bioautography is a hybrid of both previous methods [27]. In order to allow a good diffusion of the tested compounds into the agar medium, the plates can be placed in a low temperature for a few hours before incubation. The melt agar medium inoculated with microorganisms is uniformly coated on the thin plate. After the agar solidifies, the thin plate is cultured overnight at about 30 °C and dyed with a color developing agent, and then the experimental results can be observed [28]. The advantage of this method is to reduce the influence of the experimental steps on the results. The agar overlay method is suitable for broad-spectrum microorganisms, especially for yeast and bacteria. As shown in Table 1, The differences among three basic types of TLC bioautography are illustrated [29,30,31]. This method has also been well developed over the decades. For instance, Rabia Tanvir et al. applied NP silica plates to non-polar to medium-polar compounds; then, the RP silica plate was polarized [32]. In order to observe different color development patterns, the thin-layer plate was cut into two parts. Half was used for bioautography and the other half was sprayed with anisaldehyde-concentrated sulfuric acid reagent. They were observed and labeled at 365 nm and 254 nm, respectively, under UV light, and then the thin plates were coated with culture medium of inoculated *Escherichia coli*. Marzieh Sobhani et al. tested the killing ability of the extract from Plumbago europaea against *Candida albicans* ATCC 10231. After purification, the active components were characterized by one-dimensional and two-dimensional NMR, MS and UV, and the seven compounds were isolated and identified [33]. This approach will have greater potential henceforth.

### 2.2. Novel Types

#### 2.2.1. High-Performance Thin-Layer Chromatography Bioautography

HPTLC bioautography is a new technology for screening active substances, and it can provide chromatographic fingerprints that can be displayed and stored as electronic images. This technique is an improvement on the thin-layer plates, as it uses microparticle silicon (5–10 µm) with a narrow particle size distribution. The sensitivity and resolution of the thin-layer chromatography are greatly improved by using multistage or circular unfolding techniques. The major improvements of this methodology over TLC bioautography include the following: an improved resolution; a shorter analysis time; an increased detection sensitivity; and a reduced amount of developing agent, sample and culture medium.

Acacia was used to identify and quantitatively analyze multiple antibiotic residues in cow’s milk by HPTLC combined with bioautography [34]. In recent years, this method had been developed to connect with MS or NMR, and it turned out to be an advantageous combination, as it offered the possibility of a straightforward detection of bioactive analytes within the bioautography.

#### 2.2.2. D-TLC Bioautography

**Figure 2:**
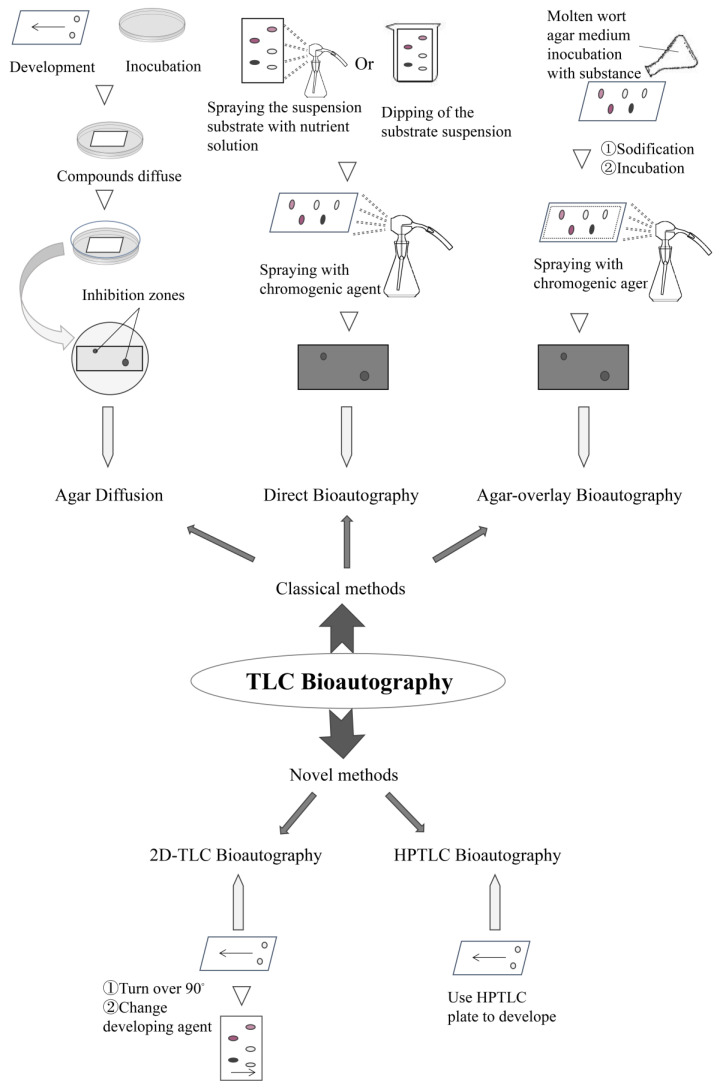
Scheme of different bioautography methods coupled with TLC. This figure introduces the main methods of TLC bioautographies and their simple operation processes. TLC bioautography is divided into two classical and novel categories. Based on the classical method, the novel methods add modern detection techniques improving the accuracy and specificity. In the agar diffusion method, the compounds transfer from the thin layer to the medium. Direct bioautography and agar overlay bioautography are detected directly on the thin layer, but there is a difference in the production of medium operation.

The first three methods must be repeated using a large number of different solvent systems to further separate both polar and non-polar chemical constituents often found in crude extracts. The 2D-TLC bioautography method remedies this defect, as the TLC plates are developed once with a polar solvent, turned 90° and then developed a second time with a non-polar solvent system. The 2D-TLC plates are then dried and sprayed with a nutrient broth. In addition, 2D-TLC bioautography eliminates the need for the development of large numbers of plates in multiple solvent systems, reduces the amount of waste solvents for disposal, and substantially reduces the time needed to identify active compounds. The technology is characterized by its ease of operation, with no need for specialized equipment, and is well suited to natural medicines with complex chemical composition. The specific operations of five types of TLC bioautography are shown in Figure 2, which is on the basis of [35].

More and more scholars are using this method to study, and, by way of illustration, HE Jia detected and tracked the antibacterial active components of *Pseudolarix kaempferi Gord* endophytic fungi by two-dimensional thin-layer bioautography and TTC staining of a *Bacillus subtilis* model [36]. David E. Wedge and Dale G. Nagle carried out two-dimensional expansion of strobilurins’ crude extracts and observed them under 254 nm UV light. After spraying, the sprays were inoculated with *Colletotrichum acutatum, C. fragariae, C. gloeosporioides* and *Phomopsis* sp. to evaluate the growth inhibition effect of fungi on the chromatographic board [37]. At the same time, this feature adds another level of high efficiency to the quantification.

## 3. Detection Technique

**Figure 3:**
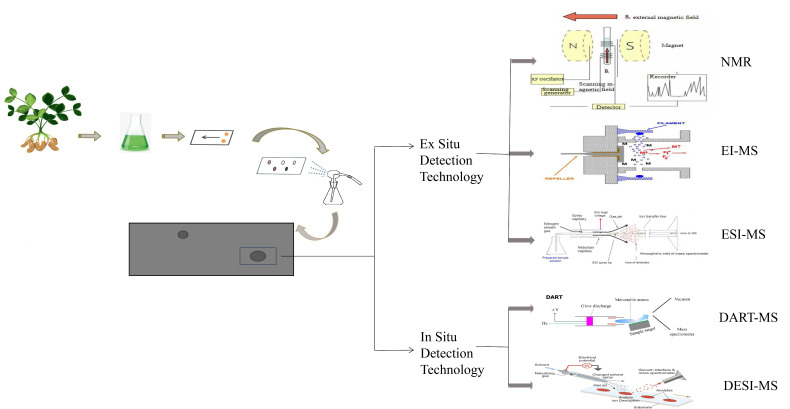
Principle of different detection methods coupled with TLC bioautography. Novel bioautography uses a variety of detection techniques, mainly including ex situ and in situ. the former mainly includes nuclear magnetic resonance (NMR), electron ionization mass spectrometry (EI-MS) and electrospray ionization mass spectrometry (ESI-MS); the latter primarily contains direct analysis in real-time mass spectrometry (DART-MS) and desorption electrospray ionization mass spectrometry (DESI-MS).

TLC bioautography can only select materials that contain active ingredients, and it is therefore crucial that follow-up tests are performed for the determination of specific components. Commonly used techniques to determine the structure of the compound technology involves ex situ and in situ techniques; the former mainly includes nuclear magnetic resonance (NMR), electron ionization mass spectrometry (EI-MS) and electrospray ionization mass spectrometry (ESI-MS), which are common detection techniques and suitable for the detection of most compounds. Traditional MS mostly uses closed ionization technology, which requires complex pretreatment operation before sample analysis, which limits the wide application of this technology. In situ ionization MS is a kind of direct ionization and mass analysis technology for samples under normal temperature and pressure conditions without sample pretreatment [38]. The advent of this technology is a major change in the field of mass spectrometry analysis, which can realize in situ, rapid, nondestructive and direct mass spectrometry analysis of trace components on the surface of objects, greatly expanding the application range of mass spectrometry analysis. In situ ionization mass spectrometry can be used to identify the structure of chemical components on the thin-layer surface; solve the problem of compound analysis after the active components are screened out by TLC bioautography; and realize in situ, real-time and direct mass spectrometry analyses of target components in spots with inhibitory effects under the background of active color on the thin layer. In particular, the direct analysis in real-time mass spectrometry (DART-MS) and desorption electrospray ionization mass spectrometry (DESI-MS) developed in recent years have attracted extensive attention for their rapid in situ analysis of samples free of preparation. In this part, utilizing systematic approaches, we summarize and discuss these detection technologies with their testing principle, their practical application, their advantages and their future development. The schematic diagram of the detection technology is shown in Figure 3.

### 3.1. Ex Situ Detection Technology

#### 3.1.1. Nuclear Magnetic Resonance

The identification of a drug candidate and its structural determination is the most important step in the process of drug discovery, and, for this, NMR is one of the most selective analytical techniques [39]. NMR refers to a spectroscopy technique based on the absorption of radio frequency radiation by the nucleus with spin properties and the generation of energy level transitions under the action of an external magnetic field. Studies have found that TLC bioautography with NMR technology has features that obtain fruitful information, penetrate into the substance without destroying it and generate highresolution images. Techniques such as 2D-NMR, 3D-NMR, gas-phase NMR and others have been developed and applied to structural identification over the years.

In order to separate the bioactive compounds from crude methanol extract of the leaf of *Excoecaria agallocha*, J. K. Patra et al. applied a technique called the agar well diffusion method on TLC and also used the HPLC and 1H NMR spectroscopy to detect the presence of compounds and possible functional groups in the crude plant extract [40]. A study about the identification of acetylcholinesterase inhibitors from galbanum used a combination of bioautography with HPTLC-MS/NMR technology. HR Adhami and others isolated the most effective and pure components [41].

#### 3.1.2. Electron Ionization Mass Spectrometry

EI-MS is the earliest ion source humans used, and it is also the first ion source used in combination with TLC bioautography; it has a simple structure, easy operation and outstanding spectral reproducibility and is still commonly used in structural analysis. Molecules are ionized and cracked by electron current bombardment to generate various positive ions with different mass-to-charge ratios, which are accelerated step-by-step by voltage and deflected by the magnetic field. Ions with different mass-to-charge ratios arrive at the collector through the slit in turn, and the mass spectrogram is obtained by amplification of the spectrogram.

For example, Prasansuklab et al. extracted poplar leaves from ethanol and divided them into neutral, acidic and alkaline components. Phytochemical components of each component were analyzed by EI-MS. At the same time, they used TLC bioautography to screen for the components with AChE inhibitory activity [42].

#### 3.1.3. Electrospray Ionization Mass Spectrometry

ESI can be applied to nonvolatile, polar and thermally unstable compounds. The scope of application is similar to TLC bioautography; thus, it is suitable to unify them. The ion source is produced via the following steps: the solution containing the components to be measured is ejected through a high-voltage tip, the charged droplets are heated to vaporize the solvent, and, finally, the components to be measured form molecular ions in the gas phase and then enter the mass analyzer for detection. ESI technology can, with the minimum of fragments of analyte, facilitate sample mixture resolution. Another advantage is that there is no need for the derivatization of analytes; therefore, a lower sample size is needed, saving analysis steps and improving detection efficiency.

It has applications in a wide range of fields. Czernicka et al. used mass spectrometry combined with liquid chromatography (LC-ESI-Q-TOF-MS) to research the qualitative and quantitative composition of obtained extracts, and the compounds were determined in two extracts according to the fragmentation pattern and existing scientific literature [43]. Recently, an ESI-based TLC-MS approach, named TLC-electrostatic field induced spray ionization (EFISI)-MS has been developed. By coupling our TLC bioautography with an in situ EFISI-MS approach, 11 known compounds with potential neuraminidase inhibitory activity were directly identified from extracts of *I. indigotica* roots [16].

### 3.2. In Situ Detection Technology

#### 3.2.1. Direct Analysis in Real-Time Mass Spectrometry

DART is based on the reaction of electrically or vibration-excited substances with reagent molecules and polar or non-polar analytes to provide better selectivity and precise elemental composition distribution through precise mass measurements [44]. In addition, the desorption-based DART-MS is applied immediately after TLC bioautography and can be easily realized by high-throughput detection and sample screening. DB-DART-MS is well suited for the analysis of a variety of small-molecule polar samples, with stable high resolution, fast real-time reaction mechanism, contactless sample analysis, solvent-free waste reduction and non-volatile chemical analysis.

DB-DART-MS was used to investigate bioactive compounds in cosmetics using the Bacillus subtilis and *Aliivibrio fischeri* bioassays for detection of Gram-positive and Gram-negative antimicrobials, respectively, and the planar yeast estrogen screen for the detection of estrogen-effective compounds [45]. Two tansy (*Tanacetum vulgare* L.) essential oils were investigated against several kinds of microorganisms, using the coupling of HPTLC-DB. The main bioactive components were identified by scanning HPTLC-DART-MS and solid-phase microextraction gas chromatography electron impact MS (SPME-GC-EI-MS) as *cis*- and *trans*-chrysanthenol as well as *trans*-chrysanthenyl acetate [46].

#### 3.2.2. Desorption Electrospray Ionization Mass Spectrometry

DESI-MS provides a method by which analytes can be directly sampled from surfaces under ambient conditions, ionized and detected using MS. As a surface sampling technique, it has proved to have great potential as a method for the readout of TLC bioautography combined with MS detection [47].

Previous work has focused on the use of DESI-MS to qualitatively identify small drug molecules and goldenseal alkaloids separated on TLC plates. In addition, DESI-MS has been shown to be suitable for quantitation of analysis separated on TLC plates [48,49]. DESI-MS is also a viable technique for the analysis of proteins, peptides and tryptic digests deposited on various types of planar media [50,51]. In short, the technology’s advantages include use without pretreatment and relatively mild and easy to direct analysis of products [52,53,54,55]. At the same time, there are some problems, such as the difficulty to ionize macromolecular samples, harsh operating conditions and inadequate research [56].

## 4. Biological Applications

TLC bioautography is a drug screening method combining the separation of TLC and the determination of biological activity. Studies have reported that this technology possesses varying levels of the capacity to verify active natural products, such as antibacterial, antifungal, antitumor and antioxidant compounds and enzyme inhibitors.

### 4.1. Screening of Antimicrobial Compounds

#### 4.1.1. Screening of Antibacterial Compounds

With the abuse of antibiotics and the increasing number of drug-resistant strains, infectious diseases and other diseases caused by pathogenic microorganisms have gradually turned into a major obstacle to the progress of human life. Therefore, searching for efficient, safe and broad-spectrum antibiotics has become a focus of attention. Antibiotics are the earliest and most widely used of the substances commonly detected, and the principle of the experiment is as follows: use the thin plate to unfold the tested object and then show the medium or make contact with the bacteria on the thin plate to locate the antibacterial components quickly and sensitively. In order to make the results easier to observe, it is sometimes necessary to add a certain amount of tetrazolium salt to the nutrient medium, such as MTT (3-(4,5-dimethylthiazol-2-yl)-2,5-diphenyltetrazolium bromide), TTC (2,3,5,-Triphenyltetrazolium chloride) or INT (iodonitrotetrazolium chloride). Dehydrogenases of living microorganisms convert these salts into colored formazans, because MTT, INT and other tetrazolium salts are light in color and easy to be converted into darker compounds by bacterial metabolites, thus forming the background color, and, as a result, for MTT, yellow zones of inhibition are observed on a purple background [57].

Early TLC bioautography was used in the study of antibiotics. In 1961, Nicholaus et al. used the agar overlay method and Brodasky et al. used the agar diffusion method to enhance the spread of antibacterial substances on a plate, resulting in a larger bacteriostatic area [58]. In 1964, Newton E et al. used a new thin-layer technique without binder adhesives to analyze the chromatograms of antibiotics, and Betina V et al., adopted *Streptococcus lactis* as an antibiotic for the detection of Gram-positive microorganisms by cultivating it on an agar surface, covering it with a TLC plate, and then adding tetrazolium salt as a color developing agent [33,59]. This technique is simple, fast and suitable for thin-layer analysis of adsorbents with or without a binder. A simple bioassay for the direct detection of antibacterial compounds on plates has been developed by Matthias O. Hamburger and Geoffrey A. Cordel [60]. A suspension of a microorganism in a suitable broth is applied to a developed TLC plate, and the bacteria are cultivated in a humid atmosphere. Then, tetrazolium salt is added to visualize the zones of inhibition. Metabolically active bacteria convert tetrazolium salt into the corresponding intensely colored formazan. Thus, antibacterial compounds appear as clear spots against a colored background. Moricz, Agnes M et al. combined OPLC with direct bioautography to study the antibacterial effects of components extracted from chamomile against two Gram-negative bacteria [61]. Ravi Ranjan Kumar et al. applied solvent extraction to extract antibacterial compounds, TLC-based bioautography to separate them and high-performance liquid chromatography (HPLC) techniques to purify the antibacterial compounds further, aiming to evaluate marine *actinomycetes* for production of antibacterial agents against pathogens [62]. We believe this application will be studied more in the coming years.

#### 4.1.2. Screening of Antifungal Compounds

The screening methods for antifungal activity in the laboratory are similar to those for antibacterial activity. The basic principles are the following: after the TLC plate is unfolded, make the plate come into contact with the fungi medium; under the appropriate conditions, no antifungal activity parts will be covered by the growth of the pathogenic microorganisms, which is in line with tetrazolium salt dying and rendering of the background color; of these, the compounds with antifungal activity will inhibit the growth of the fungi in the present inhibition spots, so as to extract them from the mixture [63].

Some studies such as that by Marzieh Sobhani et al. were aimed at finding new and more effective antifungal and antibacterial compounds against invasive vaginitis strains, separating a total of 90 extracts from 30 Iranian plant samples using *n*-hexane, ethyl acetate and methanol. Each extract was prepared in six concentrations and evaluated for antifungal activity via a micro-broth dilution method. They spotted 20 microcomponents on the thin plate and placed the ATCC 10,231 suspension of *Candida albicans* in agar medium at 50 °C and, finally, covered the thin plate uniformly, incubated it at 37 °C for 24 h and used MTT for color development [64]. L. Rahalison et al. established a simple bioautography agar overlapping method for the detection and active orientation separation of antifungal compounds using Candida albicans as an indicator and MTT assay to detect the inhibitory effect of fungal growth [65]. Later, this application led to the screening of antitumor compounds.

#### 4.1.3. Screening of Antitumor Components

A tumor is a neo-growth formed by local tissue cell proliferation under the action of various tumorigenic factors. This neo-growth is more of a space-occupying block protrusion, also known as the neoplasm. According to the cell characteristics of a neo-growth and the degree of harm to the body, the tumors typically involve benign tumors and malignant tumors that separate cancer from sarcoma, of which cancer is the most common. Malignant tumors have become an important threat to human health; therefore, research on antitumor drugs has become a hot spot. The literature shows that some scholars use TLC bioautography to screen out antitumor active substances.

For instance, Kazuo Kawasaki has built an antineoplastic activity screening system to watch the germination of *Pyricularia oryzae* P-2b or mycelia growth of abnormal morphology as an indicator. Meanwhile, Yao Xinsheng also selected some antitumor ingredients from a large number of herbal medicines with this model [66]. LJ Hanka et al. developed a quantitative microbiological assay and a bioautography system with *Penicillium avellaneum* UC-4376 for the antitumor drug maytansine and its homologues maytanprine and maytanbutine [67]. However, this method uses micro-dilution over separation, tracking and selecting; therefore, investigators cannot determine whether there are active antitumor components in crude extract samples in one step [68].

### 4.2. Screening of Antioxidant Compounds

Parts of antioxidants can be divided into the screening of DPPH, ABTS and superoxide anion free radical scavenger. DPPH radical is a synthetic, single electron, stable, nitrogen-centered paramagnetic compound. Superoxide anion radical is the first free radical produced when oxygen produces oxygen free radicals in an organism. It not only has its own toxicity but can also generate other oxygen ion free radicals through a series of reactions, causing further damage to life.

The choice chromogenic agent of the free radical scavenger is always DPPH, and its mechanism is the following: Because of its odd electron, solutions of DPPH exhibit a deep violet color. As the odd electron is paired off through the reaction with antioxidants, the violet color vanishes, and the pure yellow color caused by the picric acid group appears [9]. Thus, after the commencement of the spray on the thin-layer plate of DPPH, antioxidant activity areas are in yellow spots, while the other parts do not react, rendering the purple background. The ABTS radical reaction system also can be selected. When viewed in UV visible light or scanned at 734 nm, the plates obtain a clear blue background color, and compounds with ABTS free radical scavenging activity react with ABTS+ to produce colorless ABTS-H with white spots [69]. Corsino et al. used carotene to detect the antioxidants on the thin-layer plate. After spraying the thin-layer plate with a 0.02% β-carotene dichloromethane solution, the background color on the thin-layer plate faded under natural light, while the active part remained yellow [70]. Cespedes and Torres et al. sprayed a 0.05% β-carotene chloroform solution on the thin plate, and the same color was developed when exposed to UV rays of 254 nm [71,72].

Simulating the reaction system of xanthine and xanthine oxidase in the organism is a common method for scavenging superoxide anion free radicals. Su et al. dipped the plates into xanthine oxidase (XO) solution and then dipped the plates into a solution containing xanthine and nitro tetrachloride. The sites with O^2−^ clearing activity showed white or yellow bands, while the inactive sites showed a purple background [73]. This is a new way to screen for antioxidants.

### 4.3. Screening of Enzyme Inhibitors

#### 4.3.1. Screening of Glucosidase Inhibitors

Glucosidase is closely related to a variety of metabolic pathways and also affects islet function and lipid metabolism. Glucosidase inhibitors have consequences for controlling postprandial blood glucose, protecting the function of islet cells and improving the complications of diabetes. At present, glucosidase inhibitors have become the essential choice for the treatment of type II diabetes with poor dietary control and type Ⅰ diabetes with insulin. Therefore, the search for glucosidase inhibitors with less toxic side effects and higher efficiency from natural plants has become a hot research topic at home and abroad.

The application has been reported by several scholars such as Salazar et al. who first applied TLC bioautography technology to screen β-glucosidase inhibitors. With conjunctivitis as the substrate and FeCl3 as the staining agent, the active sites of β-glucosidase inhibitors showed white spots on the TLC plate, while the inactive sites showed a dark brown background [74]. However, this method, which uses plant extract as the enzyme substrate, has certain limitations. Later, Simespires et al. established a more specific method for the screening of α- and β-glucoside enzyme inhibitors with high sensitivity. The plant extract was spread on a TLC plate and sprayed with α- (or β-) glucosidase and then sprayed with a mixture of 2-naphthyl-α-d-glucosidase (or 2-naphthyl-β-d-glucosidase) and fast blue salt for 2–5 min to develop the color. The principle is that α-glucosidase can hydrolyze the enzyme substrate 2-naphthalyl-α-d-glucoside to release 2-naphthol, which reacts with fast blue B salt to produce purple diazo dye. On the plate, the parts with active compounds appear as white spots, while others form a purple background [75]. Yang et al. applied a combined method using Sepbox chromatography and thin-layer chromatography (TLC) bioautography to probe α-glucosidase inhibitors further. To probe active spots (or bands) on the TLC plate, the plate was first dipped in the substrate solution, dried under a stream of cold air for complete removal of the solvent and then dipped into the enzyme solution. Under the guidance of TLC bioautography, 20 compounds were successfully isolated from these fractions and 15 compounds were indicated as more potent α-glucosidase inhibitory effects than the positive control [76]. In recent years, the research on this has deepened.

#### 4.3.2. Screening of Pancreatic Lipase Inhibitors

Pancreatic lipase, also known as triacylglycerol hydrolase, is the most important enzyme for the hydrolysis of dietary fat, which can degrade 50~70% of food fat into diglycerides, monoglycerides, glycerines and fatty acids for absorption by the body. Pancreatic lipase inhibitors can impact the activity of pancreatic lipase, reduce the hydrolysis and absorption of dietary fat and have a preeminent behavior on the treatment of obesity and prevention of its complications.

On account of the inhibition of pancreatic lipase activity being one of the effective ways to prevent obesity, more and more people are paying attention to it. Hassan et al. established a TLC bioautography method for screening lipase inhibitors. This method uses α-naphthalene acetate as the substrate and fast blue B salt as the chromogenic agent. The inactive areas are purple, and the active areas are white [77]. Tang et al. developed a more stable and more specific method. This method uses β-naphthalene myristate as the substrate, which can simulate the physiological process of human beings better. They used this method to analyze the lipase inhibitor activity of six pure compounds and three crude plant extracts, among which the detection limit of orlistat was 0.01 ng, and the sensitivity was significantly improved [78].

#### 4.3.3. Screening of Tyrosinase Inhibitors

Tyrosinase is a metalloprotein belonging to the type III copper-containing protein family, which is widely distributed throughout the phylogenetic scale, from bacteria to humans [79,80]. The enzyme has unique double catalytic function leading to the resulting reactive ortho-quinones polymerise to melanin [81]. In humans, melanin is responsible for the coloring of the skin and hair, and it helps to protect the skin from the damage caused by ultraviolet radiation [82]. However, excessively high levels of melanin can cause various dermatological disorders, such as melasma, age spots and sites of actinic damage. Other diseases, including cancer and Parkinson’s disease, are also characterized by abnormalities in tyrosinase activity [83,84]. Additionally, tyrosinase takes responsibility for the enzymatic browning of fruits and vegetables during senescence or post-harvest handling and participates in wound healing, parasite encapsulation and sclerotization, playing a crucial role in developmental and defensive functions against insects [85,86]. Consequently, tyrosinase inhibitors have potential applications for improving the quality of foods, insect pest control and prevention and treatment of melanin-related health problems in humans. Thus, many scholars at home and abroad are committed to looking for a specific and efficient tyrosinase inhibitor.

Wangthong S et al. first applied TLC bioautography technology to the screening of tyrosinase inhibitors [87]. Tyrosinase and L-tyrosine were sprayed on the expanded thin plate, and the sites with tyrosinase inhibitory activity formed white spots, while the inactive sites presented a brown-purple background. However, the poor water solubility of L-tyrosine resulted in insufficient enzyme substrate and reduced detection sensitivity (the positive control was only 15.6–20.0 ng). Talbon J et al. improved the method by directly using L-dopa, which is water soluble, as the enzyme substrate and adding Triton-X100 to L-dopa additionally to avoid false positive results [88]. The conditions such as enzyme and substrate concentration, reaction temperature and time, the pH of the reaction system and the type of thin plate were optimized, and the amount of the enzyme was reduced and the detection sensitivity was improved.

#### 4.3.4. Screening of Cholinesterase Inhibitors

Cholinesterase (ChEs) play a pivotal role in regulating the signaling activity of the nervous system. There are two major types of ChEs, acetylcholinesterase (AChE) and pseudocholinesterase, or butyrylcholinesterase (BChE), and they can be distinguished both kinetically and pharmacologically [89]. Inhibiting AChE to restore acetylcholine levels is an effective treatment for senile dementia (such as Alzheimer’s disease, vascular dementia, Parkinson’s disease, or physical and cognitive symptoms associated with multiple sclerosis and Down syndrome). Among these diseases, Alzheimer’s disease (AD) is a deadly neurodegenerative disease that occurs most frequently in the elderly, which has now become one of the most serious diseases and major killers of the elderly worldwide. Current studies show that an AChE inhibitor based on the “cholinergic hypothesis” is the main drug in the treatment of Alzheimer’s disease [90]. The hypothesis suggests that the cognitive decline in at least part of patients with AD are caused by a lack of the neurotransmitter acetylcholine, thus affecting the cholinergic neurotransmission in the cerebral cortex or hippocampus, playing a vital role in memory [91]. Furthermore, plants are the main source of AChE inhibitors, and TLC bioautography technology can be used to screen substances with AChE inhibitory activity in plant extracts efficiently and rapidly.

**Figure 4:**
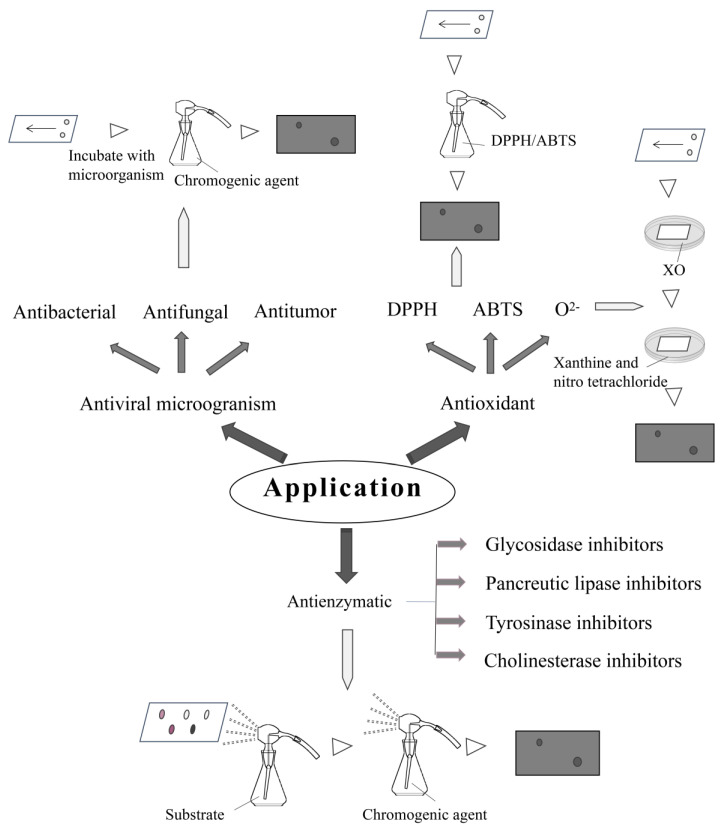
Principle of different applications in TLC bioautography. The application of TLC bioautography is mainly divided into antiviral, antioxidant and anti-enzymatic, and each item is subdivided again. This diagram illustrates the different principles of the application of this technique.

Cholinesterase inhibitors have become a hot topic due to their great influence on neurodegenerative diseases. Ellman’s method is a classical method for the determination of AchE activity; taking acetylthiocholine iodide (ATCI) as the substrate, the enzyme will decompose the substrate to thiocholine under certain pH conditions, and, after this, the thiocholine will react rapidly with the chromogenic agent in the solution producing yellow 5-thio-2-nitrobenzoic acid, which has maximum absorption at a 405 nm light wave [92]. Rhee applied the substrate and color developing agent, which were specified by Ellman’s method, to the thin plate. The part with the active compounds cannot carry out the above chemical reaction and presented white, while the other part presented light yellow on account of the inhibitory effect of the active compounds on acetylcholinesterase. Generally, the reaction should be observed within 15 min and disappear automatically after 20–30 min. The limitation of this method exists in that the background color is light, the contrast with the inhibitor spots is too small to observe and the enzyme consumption is 3 U/mL, with a high detection limit [93]. Later, Marston et al. changed the substrate and chromogenic agent and then replaced it with acetate-1-naphthalene-ester as the substrate; fast blue B salt as the chromogen, forming a purple background on the thin plate; and AChEi as a white spot [94]. The contrast becomes large and the effect changes obvious, but the deficiency lies in that the enzyme consumption is higher than the above method. The main principle of this method’s aims is that acetylcholinesterase converts acetate-1-naphthol into 1-naphthol, which can react with fast blue B salt to form a purple background while the active region shows white. The feasibility of this method was proved by the successful isolation of physostigmine with significant inhibitory activity. Based on the above, Yang et al. improved the method by reducing the amount of enzyme, the reagents and the time of the reaction. With these changes, the consumption of the enzyme was reduced by 85%, and the detection limits were decreased remarkably [95]. Anchalee Prasansuklab et al. screened the AChE inhibitory activity using a TLC-DB assay adapted from Marston’s method. This assay is based on the activity of AChE in converting the substrate 1-naphthyl acetate to 1-naphthol, which in turn reacts with the chromogenic agent fast blue B salt to produce a purple-colored diazonium dye [42]. However, the above method is only applicable to the positive phase thin plate, while the surface of the reversed phase thin plate is hydrophobic, which prevents the hydrophilic layer containing the enzyme and reagent from being deposited on its surface. In addition, the interface repulsion between the hydrophilic layer and the hydrophobic layer also reduces the diffusion velocity of the compound. Therefore, Ramallo et al. developed a bioautography system for reversed-phase laminates. Enzyme gel entrapment with an amphiphilic copolymer used for assay development not only improved the stability and reproducibility of the enzyme but also helped AchE to migrate to the non-polar inverting plate. The active spots could be detected by fluorescence and visible light with indole acetate as the substrate [96]. In addition, scholars also screened compounds of butyrylcholinesterase inhibitory activity in plants. The substrate was naphthalene acetate and the chromogenic agent was fast blue B salt. The non-inhibitory area showed a purple background due to the conversion of naphthol and fast blue B salt to a diazo compound, respectively, while the active area remained white [97]. As shown in Figure 4, the main applications and operations of TLC are described.

### 4.4. Other Applications

TLC bioautography is extensively used in various fields because of its simplicity, rapidity and agility and has gradually improved and progressed in the past decade. In addition to antibacterial, antioxidant and enzyme inhibitors, researchers are also exploring the use of TLC bioautography in other areas.

Compared with other technologies, the development time of TLC bioautography is relatively short, and there will be more growth space in the future. Okusa P. N. et al., reported a simple method for screening inhibitors of malaria pigment synthesis using thin-layer bioautography. Heme chloride and hydrochloric acid were sprayed onto the laminate and heated to initiate the formation of heme. On a brown background, the active compounds appear as distinct spots, and the experimental parameters such as reagent concentration, heating temperature, heating time and surfactant are optimized. The optimized method has been successfully applied in the detection of antimalarial drugs, inhibiting the production of hemagglutinin, chloroquine and quinine [17]. In addition, Salazar et al. used thin-layer bioautography to screen the inhibitors of the salmonella PhoP–PhoQ regulatory system [98]. Anita Surendra Patil et al. proposed a new TLC-DB method for the detection of antiurolithiatic metabolites. It helps to detect the active metabolites in P. niruri and further analyze their structures by LC-HRMS [18]. Furthermore, Zhihong Cheng et al. encourage the development of a new TLC bioautographic method for the screening of neuraminidase inhibitors to help combat influenza in an epidemic environment [16]. Okusa P. N. et al. reported a simple TLC bioautography method for screening inhibitors of malaria pigment synthesis, and later, Patil, A. S et al., proposed a novel TLC direct bioautography method for the detection of antiurolithiatic metabolites [17,18].

## 5. Conclusions

At present, the commonly used microbiological activity determination methods include the microbial inhibition method, enzymatic method, immunoassay method, radio immune assay (RIA) method, etc. These methods have their own advantages and disadvantages. For example, although the microbial inhibition method is simple in operation and low in cost, its specificity and sensitivity are not high, and it is easy to produce false positive results. However, highly sensitive, enzymatic and radio immune assays are expensive [41]. The broth micro-dilution method is a commonly used method for screening compounds with antimicrobial activity, but it needs to co-culture the compounds with the culture medium and microorganism, which takes a lot of time and narrows the scope of application, which is used to detect bacterial or fungal resistance and drug sensitivity [99,100]. Compared with the above methods, TLC bioautography is an excellent method for the determination of biological activity. This is possible with a minimum amount of laboratory equipment and apparatus, and the operation is simpler, the experimental cost lower, and the sensitivity and specificity higher, and, at the same time, the bioactivity of the metabolites or converts of the compounds can be determined by the TLC bioautography. However, TLC bioautography also has certain limitations. One such obstacle is the insensitivity of cytotoxic compounds, such as camptothecin, quassinoid and lignans. It is difficult to show the inhibitory spots on the thin-layer plate for most of these compounds. In addition, as the microbial culture medium used by the TLC bioautography is generally used as a solvent with water, the polarity is relatively large; thus, when the measured compound is too polar, it is easily spread too fast, which then leads to difficulties in the formation of inhibition spots. Questions regarding the validity of too small polarity results in compounds is a barrier to the application of this technology, because it is not easy for them to enter the medium and they also cannot form inhibition spots. This defect can be improved by the 2D-TLC bioautography established later.

It has been nearly 60 years since the development of TLC bioautography; the system is relatively mature and crucial in determining effective constituents in the field of natural pharmaceuticals, and, in particular, in this era when the “compound + biological activity” model is developing rapidly. There are several aspects for the future advancement of this technique:

To begin with, the main limitation of this technique lies in the thin-layer chromatography, while the conventional method is poor in separation. HPTLC developed subsequently uses finer and more uniform modified silica gel and cellulose as stationary phases for the hydrophobic and hydrophilic modification of the adsorbent, which can realize the separation of the positive phase and the reverse phase, improve the selectivity of the chromatography, increase the separation degree and enhance the sensitivity and reproducibility, and is more suitable for quantitative determination. OPLC refers to an elastic air cushion applied to a horizontal TLC plate; its developing agent is not forced by capillary force but by pump pressure. Therefore, the adsorbent with finer particles and a longer chromatographic plate can be used. The separation time is shortened, the diffusion effect is reduced and the separation effect is better. Micellar thin-layer chromatography (MTLC) divides into two types: normal and reverse; the developing agent of the former is with a low concentration surfactant solution on a polyamide, alumina or silica gel thin layer, and the latter is with a low concentration of non-polar organic solvent containing a small amount of water on a salinized silica gel thin layer. This method can make compounds with a similar structure that are insoluble in water achieve better separation.

Afterward, the composition complexity of natural drugs made scholars turn to in situ detection technology as the key for screening active components in the future on TLC bioautography. One of the major attractive features of these MS ion sources is the usage of direct analyte determination on sample surfaces in portable mass spectrometers, such as DESI, as they can directly detect the solid surface of the component via desorption and ionization to achieve rapid sample in situ analysis. However, the technology requires solvents and carrying remains a problem. DART replacing the solvent with inert gas has taken a step forward, which is suitable for fast portable instruments. However, after helium discharge, ions need to be separated from excited helium atoms, so the structure is relatively complex. After that, some scholars put forward a dielectric barrier discharge ion source (DBDI), which has the characteristics of a simple structure, effortless fabrication and high ionization efficiency, and it is not only suitable for application in laboratory environment but especially propitious to the miniaturization instrument [101,102]. The application of DBDI-MS for determination in multiple fields has been extensively studied in recent years. On this basis, scholars have proposed a new version of the DBDI named low-temperature plasma ion source (LTP) to produce a cold plasma at a very low temperature through a quartz tube and an electrode [103]. However, ionization is effective and visible, which is of great value in mass spectrometry and in situ biological research. Besides, the atmospheric solid analysis probe (ASAP) is an ambient ionization mass spectrometry technique developed in recent years, with a rapid (less than 30 s) identification and high-sensitivity quantitative (pg level) and accurate quality analysis, which can be performed to achieve rapid qualitative and quantitative analysis of each component in complex mixtures [104]. In addition, laser diode thermal desorption (LDTD) is a new in situ ionization technology, which is connected with MS to complete the sample analysis in a few seconds. This is suitable for liquid samples, for example, solutions, blood samples, urine samples, etc., and has been widely used in recent years [105]. It is believed that these ambient ionization techniques will combine well with TLC bioautography.

In addition to the frontier technology discussed above, broadening the application scope of TLC bioautography is, to date, the most important destination that needs to be reached. Apart from the described methods for the identification and composition screening of herbal medicines and finding new potential drugs for the treatment of serious human diseases (i.e., antibacterial components in turmeric, *Ocimum sanctum* and *Zingiber officinale* and free radical scavenger in *Piper betle* leaves and *Salvia verticillata L*.) [43,106,107,108]. TLC bioautography has a potential in other fields, such as environmental sciences (i.e., olive processing wastewaters) [109] and agriculture (i.e., pathogens related to apple ring rot disease and multiple antibiotic residues in cow’s milk) [19,34]. It can also improve the efficiency of some fermentation technologies. Therefore, wide applications of the TLC bioautography method can be expected in diversiform fields in the near future [110].

TLC has evolved over almost 100 years to supply the demand for isolation and identification. Activity studies have also been developed over the years, and, as a combination of the two, TLC bioautography technology is also constantly developing and updating. With the continuous development of science and technology and the mutual penetration of various disciplines, the expectation is that the findings of this review may also inform the design of future TLCB initiatives in the fields of drug therapy, biological studies and others. It is believed that with the joint efforts of the relevant researchers, albeit the limitations, TLC bioautography technology will be perfected, and its application prospect will be broader.

## Figures and Tables

**Table 1:** The difference between direct bioautography and agar bioautography.

Classification	Carrier	Culture Condition	Range	Sensitivity	Specificity
Agar diffusion	Culture medium	Diffusion requires incubation for several hours at 0~4 °C, and culture medium for a certain time at about 37 °C after diffusion	Suitable for broad-spectrum microorganisms	Low	Weak
Direct bioautography	Thin-layer plate	The incubation temperature is generally slightly higher than room temperature, and the incubation time is 2~3 days. It can also be cultured overnight at about 30 °C, sometimes with light	Fungal spores and certain bacteria that grow directly on thin-layer plates	High	Strong
Agar overlay bioautography	Thin-layer plate	After agar solidification, the laminates were cultured overnight at about 30 °C	Suitable for broad-spectrum microorganisms, especially for yeast, bacteria, etc.	Low	Weak

## Data Availability

Not applicable.
